# A Danger to Be Avoided

**Published:** 1877-10

**Authors:** 


A Danger to be Avoided.—A physician in Canada ordered *Hyd. Chlor.* in a prescription, which was an unpardonable blunder. The compounder put up Corrosive Sublimate, which was worse than a blunder. The patient, a lady, had a narrow escape, her life being saved by vomiting almost immediately on swallowing the poison. Again and again, while writing prescriptions containing that agent, the danger of such an error has presented to our mind. The rule should be religously observed never to abbreviate the words, but always to write in full—*Hydratis chlorali,* otherwise to put it in plain English.—*Cincinnati Medical News.*

